# Cathepsins in the Pathophysiology of Mucopolysaccharidoses: New Perspectives for Therapy

**DOI:** 10.3390/cells9040979

**Published:** 2020-04-15

**Authors:** Valeria De Pasquale, Anna Moles, Luigi Michele Pavone

**Affiliations:** 1 Department of Molecular Medicine and Medical Biotechnology, School of Medicine, University of Naples Federico II, 80131 Naples, Italy; valeria.depasquale@unina.it; 2 Institute of Biomedical Research of Barcelona, Spanish Research Council, 08036 Barcelona, Spain; ana.moles@iibb.csic.es

**Keywords:** cathepsins, mucopolysaccharidoses, lysosomal storage diseases, therapy

## Abstract

Cathepsins (CTSs) are ubiquitously expressed proteases normally found in the endolysosomal compartment where they mediate protein degradation and turnover. However, CTSs are also found in the cytoplasm, nucleus, and extracellular matrix where they actively participate in cell signaling, protein processing, and trafficking through the plasma and nuclear membranes and between intracellular organelles. Dysregulation in CTS expression and/or activity disrupts cellular homeostasis, thus contributing to many human diseases, including inflammatory and cardiovascular diseases, neurodegenerative disorders, diabetes, obesity, cancer, kidney dysfunction, and others. This review aimed to highlight the involvement of CTSs in inherited lysosomal storage disorders, with a primary focus to the emerging evidence on the role of CTSs in the pathophysiology of Mucopolysaccharidoses (MPSs). These latter diseases are characterized by severe neurological, skeletal and cardiovascular phenotypes, and no effective cure exists to date. The advance in the knowledge of the molecular mechanisms underlying the activity of CTSs in MPSs may open a new challenge for the development of novel therapeutic approaches for the cure of such intractable diseases.

## 1. Introduction

**Figure 1:**
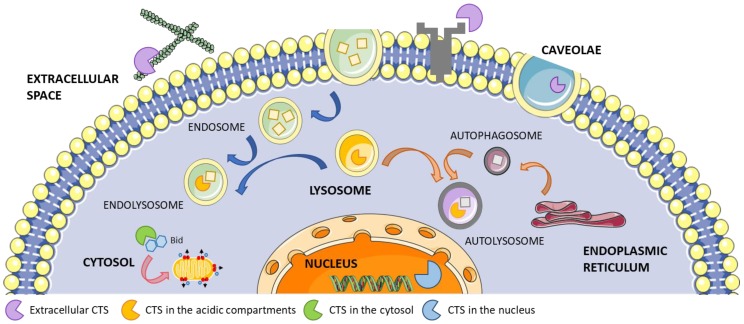
Cellular localization of cathepsins (CTSs). Here, we provide some examples of CTS functions in the different locations. CTSs in the extracellular space can cleave extracellular matrix proteins, cell receptors, or cytokines [2]. CTSs can also be found in caveolae triggering cell-surface proteolytic events associated with cell migration [3], or in the endolysosomal [4] and autolysosomal compartments where they process the compartment’s cargo [5]. CTSs in the nuclei play an important role in cell cycle regulation [6], while in the cytosol mediate mitochondrial permeabilization and apoptosis through cleavage of Bid and release of Bax [7]. Some of the cellular components displayed in the figure have been adapted from Smart Servier Medical Art under Creative Commons Attribution 3.0 Unported License.

Cathepsins (CTSs) are a family of proteases expressed in all living organisms. In humans, CTSs comprise 15 proteolytic enzymes that are classified in three distinct groups based on the key amino acid within their active site, namely serine (CTS A and G), cysteine (CTS B, C, H, F, L, K, O, S, V, X, W), and aspartate (CTS D and E) [1]. These proteases, which mostly require mild acidic conditions for their optimal activity, are all synthesized as proenzymes. Although CTSs are mainly localized in the lysosomes where the acidic environment facilitates their proteolytic activity, they are also found in the cytoplasm, nucleus, and extracellular space where they participate in extracellular matrix (ECM) protein degradation, cell signaling, protein processing, and trafficking through the plasma and nuclear membranes and between intracellular organelles (Figure 1) [2,3,4,5,6,7]. While some CTSs are ubiquitously expressed in the whole body, some are expressed in a more restricted pattern, suggesting specific cellular functions for distinct CTSs.

CTSs have been shown to play essential roles in coagulation, digestion, hormone liberation, adipogenesis, peptide synthesis, immune response, and many other vital processes [1,8]. Abnormal expression and/or activity of CTSs have been associated with a variety of human diseases, including inflammatory and cardiovascular diseases, neurodegenerative disorders, diabetes, obesity, cancer, kidney dysfunction, and many others (Table 1).

The activity and stability of CTSs are tightly regulated by glycosaminoglycans (GAGs), a class of linear, negatively charged polysaccharides that comprise the non-sulfated hyaluronic acid (HA) and the sulfated chondroitin sulfate (CS), dermatan sulfate (DS), keratan sulfate (KS), heparin and heparan sulfate (HS). The protease–GAG interactions may enable autocatalytic activation of CTSs, promote conformational changes in the CTS structures that may increase their affinity for the substrate, thus enhancing their biological activity, and finally, protect proteases from alkaline pH-induced inactivation [101,102,103].

Most of the GAGs are covalently attached to a core protein, forming proteoglycans that are abundantly found at the cell surface and ECM [104,105]. Accumulation of undigested GAGs occurs in the lysosomes as well as on the cell surface and ECM in patients affected by Mucopolysaccharidoses (MPSs). These are a group of lysosomal storage diseases (LSDs) caused by mutations in genes encoding for lysosomal enzymes involved in GAG degradation [106]. Seven types of MPSs (I, II, III, IV, VI, VII and IX) are known to differ in the type of the accumulated GAG, their prevalence and the severity of the clinical manifestations [106,107,108]. The patients exhibit neurological disorders, skeletal and joint defects, hearing and vision impairment as well as cardiovascular and respiratory disease, and premature death [108]. Current therapeutic options available for MPSs include enzyme replacement therapy (ERT), hematopoietic stem cell transplantation (HSCT), substrate reduction therapy (SRT), chaperone therapy, and gene therapy [109,110]. Despite a definite improvement for some clinical manifestations, most of these therapies are not curative, but they only ameliorate some symptoms of the disease. Indeed, the treatment of neurological disorders, avascular cartilage lesions, and cardiac dysfunctions in MPS patients still represents an unmet clinical need [110].

In this review, we summarized the current knowledge about four LSDs due to CTS gene mutations, namely galactosialidosis, neuronal ceroid lipofuscinoses (NCLs) type 10 and type 13, and pycnodysostosis. More importantly, we highlighted the involvement of CTSs in the physiopathology of MPSs that has been scarcely considered thus far, and finally, we reviewed the various types of CTS inhibitors currently available for therapeutic applications. A deeper understanding of the molecular mechanisms underlying the role of CTSs in the onset and progression of MPSs may provide a new basis for the development of novel approaches for the treatment of such diseases.

## 2. Cathepsin Deficiency Causing Lysosomal Storage Diseases

Lysosomal storage diseases (LSDs) are a family of about 70 disorders caused by disruption of lysosomal homeostasis due to inherited gene mutations. A common feature for all LSDs is the abnormal storage of macromolecular substrates or monomeric compounds inside the endosomal/lysosomal compartment. LSDs are caused by both deficiency of lysosomal enzymes and defects in non-enzymatic soluble lysosomal proteins; however, the former accounts for most LSDs [111]. LSD combined incidence is estimated at 1:5000 live births [112]. The degree of protein function, the biochemistry of the stored material, and the affected cell type determine the clinical onset and symptoms exhibited by LSD patients [113].

To date, four LSDs are known to be caused by inactivating mutations in CTS genes. Defects in CTSA, CTSD, CTSF, and CTSK result in galactosialidosis, neuronal ceroid lipofuscinoses (NCLs) type 10 and type 13, and pycnodysostosis, respectively [13,43,114,115,116,117,118,119,120,121,122,123,124,125,126,127,128,129,130,131,132,133,134,135,136,137,138,139,140,141,142,143,144,145,146,147,148,149] (Table 2).

Galactosialidosis (GSL, OMIM ID: 256540) is an autosomal recessive LSD caused by mutations in the gene encoding CTSA [13,114,115,116,117,118]. The protease CTSA, also known as protective protein/cathepsin A or PPCA, is a serine carboxypeptidase essential in beta-galactosidase (β-GAL) and neuraminidase-1 (NEU1) protein complex stabilization. Indeed, CTSA plays a protective role by preventing β-GAL and NEU-1 lysosomal degradation, although its catalytic activity is distinct from its protective function towards β-GAL and NEU1. Defects in CTSA expression and/or activity result in a complete or partial deficiency of NEU-1 and β-GAL enzyme activities, leading to the accumulation of sialylated oligosaccharides and glycoproteins in lysosomes, and excretion of the formers in body fluids. Moreover, CTSA participates in processing vasoactive peptides and the formation of elastic fibers [119].

The multiple biological functions of CTSA translate into a broad spectrum of clinical manifestations in patients affected by GSL, which are currently classified in three different forms: early infantile, late infantile, and juvenile/adult form [43]. Recurrent clinical features in the three GSL types include coarse facies, hepatosplenomegaly, dysostosis multiplex, growth retardation associated with muscular atrophy, heart involvement with cardiomegaly and thickening of the mitral and aortic valves, hearing loss, and neurological disorders [43,120,121]. To date, 28 different CTSA gene mutations have been linked to GSL, including deletions, splicing, and missense mutations [114,115,116,117,118]. Only one mutation (p.Val150Met) has been predicted to affect CTSA catalytic function, while the others are thought to most likely affect protein stability and folding [114]. Further investigation needs to clarify the link between the different mutations and the effect over CTSA, whether functional or structural. There are no effective treatments currently available for GSL patients other than supportive care.

Neuronal ceroid lipofuscinoses (NCLs), also known as Batten disease, are a clinically genetically heterogeneous group of neurodegenerative LSDs [43,121]. All NCL phenotypes exhibit early impairment of the vision, progressive decline in cognitive and motor functions, dementia, epilepsy, seizures, and, ultimately, premature death. At the cellular level, NCLs show intracellular accumulation of ceroid lipofuscins in the neurons of the central nervous system (CNS), resulting in different degrees of neurodegeneration [122]. Various types of NCLs are known due to over 430 mutations in 14 different genes (called CLNs), and they are classified into four groups based on the protein that the gene encodes such as soluble and transmembrane proteins localizing to the endoplasmic reticulum or the endosomal/lysosomal compartment [123]. CLN10 and CLN13 are included into the NCL-related group due to mutations in the genes that encode lysosomal soluble proteins/enzymes [43].

In particular, CLN10 (OMIM ID: 610127) is caused by mutations in the CTSD gene due to autosomal recessive inheritance [42]. According to the ClinVar Database, 21 mutations have been identified related to CLN10 and affecting the CTSD gene; they include 19 single nucleotide variants, one insertion, and one duplication. Among the 21 mutations, only nine mutations have been confirmed to be pathogenic and linked to the development of CLN10: six missense mutations (p.Phe229Ile, p.Trp383Cys [42], p.Gly149Val, p.Arg399His [124], p.Ser100Phe [125], p.Glu69Lys [126]), a nonsense mutation (c.764dup, p.Tyr255Ter [127]), an insertion (c.268_269insC, p.Gln90fs [128]), and one deletion (p.Phe229del [129]). All different mutations result in neuropathogenesis whose extension is determined by the degree of CTSD gene function loss. Therefore, while complete loss of CTSD activity translates in an early infantile form of CLN10 with patients dying within hours to weeks after birth, patients with residual CTSD activity develop late infantile, juvenile, or adult CLN10 with milder phenotypes [43,121,124]. It is not yet clear how CTSD deficiency causes neuropathies; however, experimental evidence suggests defective autophagy might be in part responsible, as CTSD is essential in degrading cellular components during macroautophagy [130]. Several papers have demonstrated a contribution of glial dysfunction and the involvement of various brain regions in the pathogenesis of NCLs [131]. A recent study has revealed a previously unrecognized role for CTSD in selectively modulating inhibitory synaptic vesicle trafficking and synaptic transmission, showing mechanistic evidence that GABAergic presynaptic endosomal dysfunction might account for the synaptic pathology observed in CTSD deficiency-related NCL diseases [132]. Enzyme replacement therapy (ERT) with recombinant pro-CTSD corrects defective proteolysis and autophagy in cellular and murine models of CNL10 [133]. However, to date, no therapy exists for the disease.

Mutations in the CTSF gene result in NCL type 13 (CLN13, OMIM ID: 615362), an adult-onset form of NCL, also known as type B Kufs disease [43,134,135,136,137]. Patients with CLN13 exhibit mental and motor deterioration in late adulthood [51]. To date, nine mutations with recessive inheritance are known to cause CLN13: six missense mutations (p.Gln321Arg, p.Gly458Ala, p.Ser480Leu, p.Tyr231Cys, p.Ile404Thr, and p.Cys326Phe), a nonsense mutation c.416C > A (p.S139*), a frameshift mutation (p.Ser319Leufs*27), and a mutation preventing the correct splicing of CTSF mRNA (c.213 + 1G>C) [134,135,136,137]. It has been shown that disease-causing CTSF mutants fail to cleave the lysosomal integral membrane protein type-2 (LIMP-2/SCARB2) required for normal biogenesis and maintenance of lysosomes and endosomes [138,139]; however, the exact mechanism by which CTSF deficiency translates in the clinical onset of CLN13 remains elusive. The biochemical and molecular mechanisms underlying NCLs have not been addressed yet. However, several cellular and animal models [140] of the diseases provided useful tools to study the pathogenesis of such devastating neurological disorders and to test novel therapeutic approaches as well [51].

Pycnodysostosis (PKND, OMIM ID: 265800) is an autosomal recessive LSD mainly affecting skeletal structures caused by mutations in the gene encoding CTSK [43,72,141,142]. To date, 48 different CTSK mutations have been reported including missense, nonsense, frameshift, splice-site mutations, and small insertions and deletions [43,72,142]. All the genetic modifications result either in the complete loss of the protein, defective folding and impaired enzyme activity, or faulty intracellular trafficking of the enzyme to the endo/lysosomal compartment. PKND is a specific form of osteopetrosis (increased bone density), and patients exhibit decreased bone resorption resulting in osteosclerosis, without affecting bone formation [141]. Thus, in patients with PKND, markers of bone formation such as type I collagen carboxy-terminal propeptide and osteocalcin are normal, whereas markers of bone resorption (cross-linked N- and C-telopeptides of type I collagen) are significantly decreased [143]. In vitro studies showed that mutant CTSK proteins do not degrade type I collagen, which constitutes 95% of the organic bone matrix [144]. Moreover, osteoclasts and fibroblasts from PKND specimens showed accumulation of undigested collagen fibrils in their endosomal/lysosomal compartments, reflecting the defective bone reabsorption [145]. Almost half of PKND patients have growth hormone deficiency with pituitary hypoplasia and low serum insulin-like growth factor-1 (IGF-1) levels [146,147]. However, opposite findings have been reported in in vitro cell studies using CTSK inhibitors in osteoclasts, demonstrating an increase in IGF-1 due to an impairment in the degradation of the bone matrix-secreted IGF-1 [148]. This paradox could be partially explained because PKND patients present defective osteoclastic resorption, which is responsible for the release of bone matrix embedded IGF-1. Pycnodysostosis does not correlate with increased mortality; however, it can cause significant morbidity such as recurrent fractures, osteolysis of the distal phalanges, craniosynostosis, respiratory sleep disorders, short stature, and dental problems [43]. To date, no therapy is effective for the cure of PKND, although growth hormone treatment has been shown to improve growth rates and final heights in patients with PKND [149]. Targeted enzyme or gene replacement therapies are being investigated for the cure of PKND [43].

## 3. Cathepsin Involvement in the Pathophysiology of Mucopolysaccharidoses

In MPSs, the lysosomal accumulation of undigested GAGs is considered the “primum movens” of the subsequent functional cell impairment; however, evidence demonstrates that the accumulation of storage material does not occur only in the lysosomes, but also on the cell surface and ECM where GAGs form proteoglycans through their covalent binding to a core protein [105,106,109]. The accumulation of storage material in non-lysosomal compartments accounts for impaired cell signaling and trafficking, protein unfolding, abnormal autophagy, alterations of intracellular calcium homeostasis, lysolipid accumulation, and modifications in other cellular processes that ultimately lead to the MPS phenotypes [150,151,152,153,154,155,156,157,158,159].

A variety of evidence demonstrate that abnormal expression and/or activity of both lysosomal and extra-lysosomal CTSs correlate with MPS major clinical manifestations such as neuropathology, bone and joint defects, and cardiovascular disorders (Table 3).

In particular, the cysteine CTSB, involved in the degradation of collagen [176] and responsible for heart dilatation [19], displayed a marked increase of its activity in MPS I mouse model, suggesting that the progressive heart failure and valve disease observed in these mice may be dependent on CTSB overexpression [160]. The in vivo treatment of MPS I mice with a CTSB inhibitor reduced aortic dilatation and heart valve thickening, and led to an improvement of cardiac function, suggesting that CTSB inhibition may have a potential benefit in the disease [161]. Elevated activity of CTSB was detected in the MPS VII dog model showing abnormalities in the collagen structure of mitral valve [162]. When affected dogs received an intravenous injection of a retroviral vector expressing canine β-glucuronidase (the deficient enzyme in MPS VII), a reduced CTSB activity was observed, which correlated with an improved signal for structurally intact-collagen. Furthermore, in both mouse and dog models of MPS I and MPS VII, aortic dilatation resulted in being associated with an up-regulation of the elastase CTSS as well [171,172,173]. Indeed, neonatal intravenous injection of a retroviral vector expressing α-L-iduronidase normalized CTSS mRNA levels and prevented aortic disease in MPS I mice [171]. Moreover, intravenous injection of a retroviral vector expressing β-glucuronidase to MPS VII dogs reduced RNA levels of CTSS and delayed the development of aortic dilatation [173].

Cardiac involvement, although firstly reported for MPS I, II, and VI affected patients, has been reported in all MPS patients [177]. Indeed, cardiac disease has been described in MPS III patients as well as in patients affected by the other MPS subtypes [178,179]. Multiple evidence demonstrated the involvement of CTSs in many cardiovascular diseases, including atherosclerosis, cardiac hypertrophy, cardiomyopathy, myocardial infarction, and hypertension, some of which are common clinical manifestations in MPSs [8,10,17,18,19,36,56,61,66,76,80,81]. In particular, CTSB results in being up-regulated in cardiomyocytes in response to hypertrophic stimuli both in vivo and in vitro [180]. Furthermore, CTSB was associated with an increased risk of cardiovascular events in patients with stable coronary heart disease [18]. A specific CTSB inhibitor, namely CA-074Me, reduced cardiac dysfunction, remodeling, and fibrosis in a rat model of myocardial infarction [181]. Studies aimed to explore the potential of CTS inhibitors for the treatment of cardiovascular diseases are ongoing [19], and targeting CTSs–based therapy might provide new avenues for the treatment of MPSs as well.

Beside cardiac disease, some MPS subtypes are characterized by central nervous system (CNS) degeneration for which there are currently no resolutive treatments [106,109]. The involvement of CNS in the disease manifests as mental retardation, intellectual disabilities, behavioral disorders, sleep disturbances, progressive neurodegeneration, and early death. Neurodegeneration occurs in the severe forms of MPS I, and it is prominent in MPS III affected patients [106,108,182]. Elevated transcripts of CTSD, CTSS, and CTSZ were detected in the cortex of MPS I and MPS IIIB mouse models [166]. Abnormal activity of CTSD in the cerebral cortex correlated with locomotion disorders and neuropathology in MPS I mice [167]. Up-regulation of CTSB was observed in the brain of MPS IIIA mice [163]. Overexpression of CTSB and CTSD was also found in the proteomic profile of MPS I mouse brain tissues [164]. Interestingly, enhanced expression and activity of CTSB resulted in being associated with increased deposition of amyloid plaques in the MPS I mouse brain, and the existence of a novel CTSB-associated amyloidogenic pathway leading to neurodegeneration was highlighted [165]. Since CTSB is a crucial regulator of the NLRP3 inflammasome, it likely contributes to the inflammasome-dependent pathway involved in MPS neuroinflammation [183]. On the other hand, the cysteine CTSB and the aspartate CTSD result to be up-regulated in a variety of neurological disorders [184,185,186]. The protease CTSS is preferentially expressed in cells of the macrophage/monocyte lineage, and inflammation stimulates its secretion from the microglia and macrophages [185,187]. The involvement of microglial CTSB, CTSD, and CTSS in neurodegenerative diseases supports the view that microglia-driven neuroinflammation contributes to the progression of neurodegeneration in MPS I and IIIB [183,188,189]. Indeed, molecular evidence of microgliosis has been well established in mouse and dog models of MPS I and MPS III A, B, and C subtypes [166,182,188,190]. Inhibition of CTSB has been shown to prevent neuronal death and behavioral disorders in a patient affected by the Niemann-Pick disease type A and in a mouse model of the disease [191]. Although further studies are needed to fully elucidate the pathophysiological role of CTSs in the CNS, the above findings strongly suggest that the specific inhibition of microglial CTSs might lead to neuroprotective outcomes in MPS phenotypes characterized by activated pro-inflammatory microglia.

Neurological phenotypes are also common in other MPS types than MPS I and III. Indeed, patients affected by the severe forms of Hunter syndrome (MPS II), which account for about 75% of the cases, exhibit impairment of cognitive skills, mental retardation, intense neurobehavioral symptoms, and death in the second decade of life [192]. In agreement with the previous findings in MPS I and III subtypes, a transcriptome analysis of the brain from the MPS II mouse model showed CTSD up-regulation in the cerebral cortex of affected mice [170]. The same study also highlighted dysregulation of CTSA, CTSC, CTSH, CTSL, and CTSS gene expression in MPS II mouse brain, although with different trends (up/down-regulation) in the cerebral cortex and the midbrain/diencephalon/hippocampus areas. Variation in CTS gene expression between different brain regions was also observed in the MPS VII mouse model, thus suggesting that different neuropathologic mechanisms may predominate in the different areas of brains [174]. Furthermore, a transcriptome analysis of MPS VII mouse brain showed that CTSA, CTSB, CTSC, CTSD, CTSH, CTSS, and CTSZ were highly up-regulated in all brain regions, while CTSK was only changed in the brain stem and was down-regulated. Integrated analysis of proteome and transcriptome changes confirmed CTSS and CTSZ dysregulation in the MPS VII mouse hippocampus [175]. MPS VII affected patients present a broad clinical spectrum of symptoms from severe to milder phenotypes; however, most of them display intellectual disabilities together with delayed speech development, hearing impairment, and behavioral disturbances [193].

The accumulation of undigested GAGs in the lysosomes of connective tissue cells and chondrocytes is responsible for musculoskeletal abnormalities commonly observed in almost all MPS subtypes [100,101,102,103,104,105,106,194,195,196]. However, in MPSs, the pathogenesis of the skeletal and joint disease, including growth impairment, may involve complex molecular mechanisms underlying alterations of cartilage and bone metabolism, as well as inflammatory pathways [197]. Indeed, metabolic inflammation is a significant cause of osteoarticular symptoms in MPS disorders [183,198]. On the other hand, CTSs have long been involved in skeletal and bone health and disease [199]. Recently, up-regulation of CTSA, CTSH, and CTSZ has been detected through transcriptomic and proteomic analyses in a rat model of spinal cord injury [200]. In VCP (valosin containing protein) knock out mice, up-regulation of CTSB and CTSD in skeletal muscle correlated with activation of the transcription factor EB [201]. The cysteine protease CTSK, which has long been known as a molecular marker of differentiated osteoclasts and is directly involved in the degradation of bone matrix proteins [201,202,203], plays a crucial role in skeletal pathologies frequently observed in MPSs and other LSDs as well [168]. In the murine model of MPS I, the accumulation of GAGs in bones had an inhibitory effect on CTSK activity, resulting in impaired osteoclast activity and decreased cartilage resorption, thus contributing to the bone pathology seen in the disease [169]. This finding makes CTSK a candidate therapeutic target for MPS types where current therapies have a limited effect on skeletal conditions.

## 4. Cathepsin Inhibitors and Their Therapeutic Applications

The inhibition of CTSs has been widely explored over the last decades in the field of chronic inflammatory diseases [27,37,204,205], cardiovascular diseases [10,19,181], osteoporosis [70,71,72,73], arthritis [28,206], kidney diseases [30,31,32,84], pancreatitis [207], obesity [208,209,210], cancer [25,34,48,74,82,211], neurodegenerative diseases [39,41,184,185,212,213], and many other pathological states. Multiple inhibitors are currently available, ranging from reversible covalent inhibitors to irreversible inhibitors [214,215,216,217,218] (Table 4).

The class of reversible inhibitors acts by engaging the target protein through non-covalent interactions, whereas irreversible inhibitors bind the target protein with stable covalent bonds [219]. Although both types of CTS inhibitors have shown to work efficiently, reversible inhibitors have been proved to exhibit higher selectivity [216,218,219,220]. Moreover, off-target effects with broad-spectrum inhibition of proteases, leading to unpredictable side effects in clinical trials, has represented the main concern for the therapeutic use of CTS inhibitors in humans. The recent focus on target- and ligand-binding drug design to selectively inhibit specific CTSs has provided excellent results in overcoming such issue [19,72,204,205,206,214,215,216,217,218,219,220,221,222]. In this context, several new strategies have been reported for successfully CTS targeting, including designed ankyrin repeat proteins (DARPins) with high CTSB blocking activity [222], non-peptide synthetic molecules with anti-CTSK [71] and anti-CTSD [223] activity, and naturally occurring asperphenamate [224]. Natural depsipeptides inhibitor of CTSD, namely izenamides A, B, and C, have been recently successfully tested [225], and quantum mechanics/molecular modeling studies of the mechanism of cysteine protease inhibition by dipeptidyl nitroalkenes provided promising results [226].

Selective CTSG inhibitors have been recently designed with the potential to improve chronic inflammatory diseases [205]. A variety of CTSC inhibitors have been developed and evaluated in preclinical/clinical trials to regulate serine protease activity in inflammatory and immunologic conditions [27,28]. Pepstatin A is a potent inhibitor for CTSD [227]. Although Pepstatin A can target other aspartyl proteases than CTSD, it is 26,000 times more specific for CTSD (Ki = 0.5 µmol/L) than for its next target renin (Ki = 13000 µmol/L). Pepstatin A has been proved effective in slowing down chronic kidney disease progression [30,32] and fatty liver disease [209] in experimental animal models. Due to the low bioavailability of this peptidic inhibitor, efforts have been made to design Pepstatin A analogues more suitable for the treatment of human diseases [228]. Highly specific and potent small-molecule inhibitors of CTSD other than Pepstatin A have been developed for the treatment of non-alcoholic fatty liver disease [229], as well as CTSD targeting by natural products has shown to be beneficial in cancer chemoprevention [216].

The aspartic protease CTSE plays an essential role in antigen processing within the class II MHC pathway [48], therefore broadly inhibiting CTSE can lead to undesirable side effects. Selective inhibitors of CTSE and CTSB, both involved in the polarization of microglia/macrophages in neurotoxic phenotypes leading to hypoxia/ischemia, are being tested as pharmacological agents for the treatment of ischemic brain injury [212]. Moreover, CTSS inhibitors have shown neuroprotective and anti-inflammatory effects in preclinical studies for the treatment of neurodegenerative diseases [184], although CTSS essential role in CNS homeostasis might limit its therapeutic applications [204]. However, molecular modeling-assisted design of CTSS inhibitors has provided novel scaffolds for improved CTSS inhibition [217]. Experimental evidences suggest that inhibition of CTSS attenuates the progression of atherosclerosis during chronic kidney disease [84], improves sugar levels during type2 diabetes [208], and prevents autoantigen presentation and autoimmunity [229]. CTSK inhibitors have been proved successful improving osteoporosis [72,73,230]; however, concerns emerged over off-target effects of the inhibitors against other CTSs and CTSK inhibition at nonbone sites (i.e., skin, and cardiovascular and cerebrovascular sites). Recently, novel selective inhibitors for CTSK have been developed, showing beneficial effects on bone and cartilage in preclinical osteoarthritis models with a safety profile [71,206].

The use of CTS inhibitors in cellular and animal models have contributed to deepening our understanding of the mechanisms of action and biological functions of these proteolytic enzymes.

## 5. Conclusions

Neuropathology, skeletal and joint defects, and cardiac disorders are among the most prominent clinical manifestations of MPSs, which are refractory to the current therapies [106,108,109]. Although no studies are available in humans, investigations in animal models have shown a beneficial effect of CTS inhibition, especially in ameliorating cardiac disease in MPSs and other LSDs [161,162,171,173]. Since CTSs have also been shown to play a role in the onset and progression of neuropathology and skeletal disorders in MPSs, affected patients might gain benefits from treatments with CTS targeting-based drugs. Therefore, it would be of great interest to test the effectiveness of the new generation of highly selective CTS inhibitors in MPSs. They could be used alone or in combination with the current therapeutic approaches to improve the quality and duration of life of these patients. However, there is a need for further investigations on the effective role of distinct CTSs in the pathophysiology of MPSs to recognize them as key players in the fight against such incurable diseases.

## Figures and Tables

**Table 1:** Cathepsin-related diseases.

Cathepsin (CTS)	Type	MEROPS ID [9]	Disease	References
CTSA	Serine	S10.002	Cardiovascular disease	[10,11]
			Charcot-Marie Tooth diseases	[12]
			Galactosialidosis	[13]
			Muscular dystrophy	[14]
CTSB	Cysteine	C01.060	Alzheimer’s disease	[15]
			Cancer	[16]
			Cardiovascular disease	[17,18,19]
			Liver fibrosis	[20,21]
			Pancreatitis	[22,23]
CTSC	Cysteine	C01.070	Cancer	[24,25]
			Inflammatory/autoimmune diseases	[26,27,28]
			Papillon–Lefèvre syndrome	[29]
CTSD	Aspartate	A01.009	Acute and chronic renal disease	[30,31,32]
			Cancer	[33,34,35]
			Cardiac disease	[36]
			Chronic obstructive pulmonary disease	[37]
			Gaucher disease	[38]
			Liver fibrosis	[21]
			Neurodegenerative disorders	[39,40,41]
			Neuronal ceroid lipofuscinosis 10	[42,43]
			Niemann-Pick type C	[44]
			Pancreatitis	[23]
CTSE	Aspartate	A01.010	Chronic obstructive pulmonary disease	[37,45]
			Neuropathies	[46,47]
			Pancreatic cancer	[48]
CTSF	Cysteine	C01.018	Alzheimer’s disease	[49]
			Cancer	[50]
			Neuronal ceroid lipofuscinosis 13	[43,51]
CTSG	Serine	S01.133	Autoimmune diseases	[52,53]
			Cystic fibrosis	[54]
			Chronic obstructive pulmonary disease	[37,55]
			Coronary artery disease	[56]
			Inflammatory bowel disease	[57]
			Pancreatitis	[58]
			Psoriasis	[59]
			Rheumatoid arthritis	[60]
CTSH	Cysteine	C01.040	Aortic aneurism	[61,62]
			Myopia	[43,63]
			Narcolepsy	[64]
			Type I diabetes	[65]
CTSK	Cysteine	C01.036	Aortic aneurism	[61,62]
			Atherosclerosis	[66]
			Cancer	[67]
			Chronic obstructive pulmonary disease	[37]
			Lung fibrosis	[68]
			Osteoarthritis; Osteoporosis	[69,70,71,72,73]
			Pycnodysostosis	[43,73]
CTSL	Cysteine	C01.032	Cancer	[74,75]
			Cardiovascular disease	[19,61,62,76]
			Chronic kidney disease; Diabetic nephropathy	[77,78]
CTSO	Cysteine	C01.035	Unknown	
CTSS	Cysteine	C01.034	Alzheimer’s disease	[79]
			Atherosclerosis	[18,19,80,81]
			Cancer	[82]
			Chronic obstructive pulmonary disease	[37]
			Chronic kidney disease	[83,84]
			Gaucher disease	[38]
			Metabolic syndromes	[85]
CTSV	Cysteine	C01.009	Atherosclerosis	[81,86]
			Cancer	[87,88,89]
			Hyperhomocysteinemia	[90]
			Myasthenia gravis	[91]
			Sickle cell disease	[92]
CTSW	Cysteine	C01.037	Gastritis	[93,94]
			Leukaemia	[95]
CTSX/Z	Cysteine	C01.013	Ageing and neurodegeneration	[96]
			Cancer	[88,97,98,99]
			Helicobacter pylori	[14,100]

**Table 2:** Lysosomal storage diseases caused by cathepsin mutations.

LSD Type	OMIM ID	Gene Deficiency	Biological Effect	References
Galactosialidosis	#256540	CTSA	Accumulation of sialylated oligosaccharides and glycoproteins in lysosomes due to complete deficiency in neuraminidase-1 and partial deficiency in beta-galactosidase.	[13,43,119,121,122]
Neuronal ceroid lipofuscinosis 10	#610127	CTSD	Impaired lysosomal degradation and aberrant autophagy.	[42,43,130,131]
Neuronal ceroid lipofuscinosis 13	#615362	CTSF	Impaired lysosomal degradation and aberrant autophagy.	[43,44,45,46,47,48,49,50,51,52,53,54,55,56,57,58,59,60,61,62,63,64,65,66,67,68,69,70,71,72,73,74,75,76,77,78,79,80,81,82,83,84,85,86,87,88,89,90,91,92,93,94,95,96,97,98,99,100,101,102,103,104,105,106,107,108,109,110,111,112,113,114,115,116,117,118,119,120,121,122,123,124,125,126,127,128,129,130,131,132,133,134,135,136,137,138,139,140]
Pycnodysostosis	#265800	CTSK	Impaired osteoclast-mediated bone resorption.	[43,141,142,145]

**Table 3:** Cathepsin involvement in mucopolysaccharidoses (MPS).

MPS Type	OMIM	Cathepsin	Pathological Effect	References
**MPS-I** (Hurler Syndrome)	607014 607015 607016	CTSB	Cardiac disease	[160,161,162]
		CTSB	Neurological disorders	[163,164,165]
		CTSD	Neurological disorders	[164,166,167]
		CTSS, CTSZ	Neurological disorders	[166]
		CTSK	Skeletal disorders	[168,169]
**MPS-II** (Hunter Syndrome)	309900	CTSA, CTSC, CTSD, CTSH, CTSL, CTSS	Neurological disorders	[170]
**MPS-III** (Sanfilippo Syndrome)	252900 252920 252930 252940	CTSD, CTSS, CTSZ	Neurological disorders	[166]
		CTSK	Skeletal disorders	[168]
**MPS-IV** (Morquio Syndrome)	253000	CTSK	Skeletal disorders	[168]
**MPS-VI** (Maroteaux-Lamy Syndrome)	253200	CTSK	Skeletal disorders	[168]
**MPS-VII** (Sly Syndrome)	253220	CTSS	Cardiac disease	[171,172,173]
		CTSA, CTSB, CTSC, CTSD, CTSH, CTSK, CTSS, CTSZ	Neurological disorders	[174,175]
		CTSK	Skeletal disorders	[168]

**Table 4:** Inhibitors of cathepsins.

Irreversible Covalent Inhibitors	Reversible Non-Covalent Inhibitors
Epoxysuccinates	Aldehydes and ketones
Oxiranes and strained ring electrophiles	Cyclopropenones
Michael acceptors	α-Keto derivatives
Halomethyl ketones	Nitriles
Diazomethyl ketones	
Acyloxymethyl and other activated ketones	
